# Molecular, Cellular and Physiological Evidences for the Anorexigenic Actions of Nesfatin-1 in Goldfish

**DOI:** 10.1371/journal.pone.0015201

**Published:** 2010-12-03

**Authors:** Ronald Gonzalez, Brent Kerbel, Alexander Chun, Suraj Unniappan

**Affiliations:** Laboratory of Integrative Neuroendocrinology, Department of Biology, York University, Toronto, Ontario, Canada; King's College London, United Kingdom

## Abstract

**Background:**

Nesfatin-1 is a recently discovered anorexigen encoded in the precursor peptide, nucleobindin-2 (NUCB2) in mammals. To date, nesfatin-1 has not been described in any non-mammalian species, although some information is available in the sequenced genomes of several species. Our objective was to characterize nesfatin-1 in fish.

**Methodology/Principal Findings:**

In the present study, we employed molecular, immunohistochemical, and physiological studies to characterize the structure, distribution, and appetite regulatory effects of nesfatin-1 in a non-mammalian vertebrate. A very high conservation in NUCB2 sequences, especially in the nesfatin-1 region was found in lower vertebrates. Abundant expression of NUCB2 mRNA was detected in several tissues including the brain and liver of goldfish. Nesfatin-1-like immunoreactive cells are present in the feeding regulatory nucleus of the hypothalamus and in the gastrointestinal tract of goldfish. Approximately 6-fold increase in NUCB2 mRNA levels was found in the liver after 7-day food-deprivation, and a similar increase was also found after short-term fasting. This points toward a possible liver specific role for NUCB2 in the control of metabolism during food-deprivation. Meanwhile, ∼2-fold increase at 1 and 3 h post-feeding and an ∼3-fold reduction after a 7-day food-deprivation was observed in NUCB2 mRNA in the goldfish hypothalamus. *In vivo*, a single intraperitoneal injection of the full-length native (goldfish; *gf*) nesfatin-1 at a dose of 50 ng/g body weight induced a 23% reduction of food intake one hour post-injection in goldfish. Furthermore, intracerebroventricular injection of *gf*nesfatin-1 at a dose of 5 ng/g body weight resulted in ∼50% reduction in food intake.

**Conclusions/Significance:**

Our results provide molecular, anatomical and functional evidences to support potential anorectic and metabolic roles for endogenous nesfatin-1 in goldfish. Collectively, we provide novel information on NUCB2 in non-mammals and an anorexigenic role for nesfatin-1 in goldfish.

## Introduction

Nesfatin-1 is a recently identified anorectic peptide cleaved from the N-terminal of the precursor protein nucleobindin 2 (NUCB2) [1]. Nesfatin-1 immunoreactivity (ir) has been detected in mammalian brain regions that regulate feeding and metabolism [1]–[8] and peripherally in the endocrine cells of the stomach, pituitary [9] and pancreas [9]–[11]. In addition, nesfatin-1 has been detected in both rat cerebrospinal fluid [1] and in the peripheral circulation of humans and rodents [9], [12]–[14]. Several reports provide clear evidence in support of an inhibitory role for nesfatin-1 on food intake and body weight [1], [13]–[15]. Acute administration of nesfatin-1, by central or peripheral injections has been shown to reduce food intake in rats for up to 6 hours [1], [13]–[15]. In addition, prolonged continuous central infusion of nesfatin-1 reduces subcutaneous, mesenteric and epididymal fat mass [1]. In addition to this, nesfatin-1 has been shown to regulate cardiac functions [16], reduce blood glucose levels in diabetic mice [14], and induce fear and anxiety like behaviors [17]. Thus, nesfatin-1 is emerging as a multifunctional peptide with predominant anorectic effects in mammals.

Oh-I et al. (2006) initially proposed the post-translational processing of NUCB2 (pronesfatin) by prohormone convertases (PCs) through specific cleavage sites. These cleavage sites which consist of the basic amino acid pairs, Lys-Arg and/or Arg-Arg remain conserved in goldfish. It is well known that PC1/3 and PC2 are the major PCs involved in prohormone processing. In addition, previous studies have described the presence of PC1/3 and PC2 in early evolution [18] including in fish [19], [20]. Thus, if we infer that PCs perform a similar function in non-mammalian vertebrates the processing of pronesfatin by PCs may yield nesfatin 1, nesfatin-2, and nesfatin-3, as proposed in mammals. According to Shimizu et al., (2009) the mid-segment of nesfatin-1 (24–53 amino acids) is the active appetite inhibitory segment, as intraperitoneal injections of the c- (1–23) and n-terminals (54–82) of nesfatin-1 had no anorectic effects in mammals. While the physiological roles of nesfatin-1 in mammals are being characterized, the existence and functions of NUCB2 and encoded peptides in non-mammalian vertebrates remain unexplored. We hypothesized that NUCB2 is present in lower vertebrates and nesfatin-1 has appetite regulatory effects in fish. The objectives of this study were to identify NUCB2 sequences in lower vertebrates, and to characterize the appetite regulatory effects of nesfatin-1 in goldfish, a very well characterized and widely used organism for studying feeding regulatory peptides. We identified NUCB2 mRNA sequences from many lower vertebrates, and further characterized the appetite regulatory role of this peptide using goldfish. Both NUCB2 mRNA and nesfatin-1-like ir was found in the appetite regulatory regions of the brain and also in several peripheral tissues of fish. Food deprivation caused a significant reduction in hypothalamic NUCB2 mRNA expression. Acute central or peripheral injections of the synthetic form of native nesfatin-1 inhibits food intake in goldfish. Collectively, these results provide novel evidences for an evolutionarily conserved structure and biological action for nesfatin-1 in fish.

## Materials and Methods

### Ethics Statement

All experimental protocols using fish were approved by the York University Animal Care Committee, and strictly adhered to the policies of the Canadian Council for Animal Care.

### Animals

Male and female goldfish (*Carassius auratus*; common variety) (3–4 inches long and 20–30 g body weight) were purchased from local suppliers and maintained at 20 C water temperature under a simulated natural photoperiod (10 h light:14 h dark). All fish were fed commercial pellet diet (Martin Profishent, Ontario, Canada) once a day at 12:00 PM to satiety. For all feeding studies, food intake was monitored daily during the 2-week acclimation period. Fish were anesthetized in 0.015% tricainemethane sulfonate (Syndel Laboratories, Vancouver, Canada) before intracerebroventricular (ICV), intraperitoneal (IP) injections and dissection of tissues for total RNA extraction.

### Materials

Goldfish nesfatin-1 (1–82) (*gf*nesfatin-1; VPISIDKTKVKLPEETVKES PQNVDTGLHYDRYLREVIDFLEKDQHFREKLHNTDMEDIKQGKLAKELDFVSHHVRTKLDEL) was custom synthesized by Genscript (Piscataway, NJ). The purity of the peptide was confirmed by using HPLC, Electrospray Ionization Mass Spectrometry and MALDI-TOF. The peptide was synthesized with 98% purity, with a single peak confirming the predicted mass. Peptide aliquots were always freshly reconstituted in fish physiological saline [21] prior to use.

### Identification and phylogenetic analysis of non-mammalian NUCB2 sequences

NUCB2 gene/mRNA and protein sequencing information for zebrafish (*Danio rerio*), Western clawed frog (*Xenopus tropicalis*), chicken (*Gallus gallus*), mouse (*Mus musculus*) and human (*Homo sapiens*) were obtained from annotated sequences available through the GenBank (http://www.ncbi.nlm.nih.gov/genbank/) and the Ensembl genome databases (http://www.ensembl.org/index.html). With the exception of zebrafish, all other teleost NUCB2 sequences have not yet been annotated. In addition, two paralogous zebrafish NUCB2 genes, NUCB2A and NUCB2B, exist due to the teleost-specific whole-genome duplication. We compiled all other teleost NUCB2 sequences from the Ensembl genome databases using the zebrafish NUCB2A and NUCB2B protein sequences as the *in silico* probes. We aligned similar sequences which most highly match with those two paralogous NUCB2 in zebrafish within the genomes of stickleback (*Gasterosteus aculeatus*), medaka (*Oryzias latipes*), green pufferfish (*Tetraodon nigroviridis*) and fugu (*Takifugu rubripes*). In addition, NUCB1 sequences were available for many species within Genbank and Ensembl databases. The NUCB1 and NUCB2 sequences obtained were aligned using ClustalW2 general purpose multiple sequence alignment program for DNA or protein [22]. A phylogenetic analysis based on the amino acid sequences was constructed using the freeware “Phylogeny.fr” (http://www.phylogeny.fr/) [23].

### Determination of goldfish NUCB2 mRNA

Total RNA was extracted from goldfish brain using the TRIzol® RNA isolation reagent (Invitrogen, Canada). RNA purity was checked by optical density (OD) absorption ratio (OD 260 nm/OD 280 nm) using a Multiskan® Spectrum spectrophotometer (Thermo, Vantaa, Finland). Only samples with an absorption ratio >1.7 were used for cDNA synthesis. Synthesis of cDNAs was conducted using iScript™ cDNA synthesis kit as directed by the manufacturer (BioRad, Canada). The cDNAs were used as templates for reverse transcription-PCR (RT-PCR) with two primers based on the zebrafish NUCB2 mRNA sequences available on Genbank (NM_201493): gfNes-1F and gfNes-1R (Table S1). The subsequent partial amplification (885 bp) of goldfish NUCB2 cDNA was gel extracted and purified using a QIAquick Spin kit (QIAGEN, Canada). To obtain the full-length NUCB2 cDNA sequence, the partial NUCB2 cDNA was extended using 3′ and 5′ rapid amplification of cDNA ends as directed by the manufacturer of the 3′ and 5′ RACE kits (Invitrogen, Canada). The following gene specific primers were used for 3′RACE: gfNes-3 1FP and gfNes-3 2FP, and for 5′RACE: gfNes-5 GSP1 and gfNes-5 GSP2 (**Table S1**).

### Tissue Distribution of NUCB2 mRNA in Goldfish

The following tissues were collected from goldfish: hindbrain, midbrain, hypothalamus, telencephalon, olfactory bulbs, pituitary, anterior intestine (J-loop), posterior intestine (rectum), kidney, spleen, liver, heart, eye, muscle, gill, gall bladder, adipose, ovary, testes, and skin. Total RNA was extracted and cDNA was synthesized as described above. Quantification was conducted using species-specific primers for NUCB2A, gfNes-qRTF2, gfNes-qRTR1 (**Table S1**). The gene specific primers amplified a 196 bp region within the nesfatin-1 encoding region. The amplicons spanned exons 3–5 serving as a control for genomic contamination. Based on the zebrafish paralogous NUCB2 sequences, exon 2 for NUCB2A is 20 base pairs larger than exon 2 in NUCB2B. In addition, the template products were validated by DNA sequencing performed with the Applied Biosystems DNA Sequencer (3130xL) using the BigDye® Terminator chemistry and showed the highest similarity to zebrafish NUCB2A in a BLAST search. Furthermore, subsamples of all real-time quantitative reverse transcription PCR (qRT-PCR) products were electrophoresed to confirm the amplicon size. No double band was observed suggesting the paralogous NUCB2B gene was not amplified. Species-specific β-actin primers served as internal controls to normalize cDNA quantity for each tissue sample. Quantification, using qRT-PCR, of NUCB2 and β-actin mRNA was performed in triplicate on all samples using an iQ™ SYBR® Green Supermix on a Chromo4™ Multicolor Real-Time PCR Detection System (Bio-Rad, Canada). Thermal cycling was conducted as follows: 3 minutes at 95°C, then 40 cycles were performed with 95°C denaturation step for 30 seconds, an annealing temperature of 59°C for 30 s and an extension temperature of 73°C for 30 min. To confirm the efficiency of the reactions, six 2-fold dilution standards were used in the making of a standard curve and calculations of amplification efficiency were conducted for each qRT-PCR assay. The efficiency (*E*) of PCR was calculated from the regression slope of the assay (*E = 10^-1/slope^*). REST-MCS^©^ version 2 software was used to analyze the data. Relative expression data was obtained after normalization of NUCB2 mRNA expression in each sample using β-actin expression from the same sample. Data were normalized according to the Pfaffl method [24]. Purity of each amplicon was confirmed by a melting curve, which consisted of a slow ramp from 55 C to 95 C with SYBR Green readings taken every 1 C for 40 cycles.

### Periprandial Expression of NUCB2 mRNA in Goldfish Hypothalamus and Liver

In all vertebrates the hypothalamus, adipose tissue, and liver are major regulators of food intake and energy homeostasis. In this study, we examined periprandial (pre- and post-feeding) changes of NUCB2 mRNA expression in the hypothalamus and liver, two tissues that show abundant expression of NUCB2. Seven groups of weight-matched goldfish (*n = 6/group*) were acclimated for 2 weeks to tank conditions and fed daily at a scheduled time (12:00, 0 h) for 2 weeks. Hypothalamus and liver samples were collected at: 3 h prior to feeding (−3 h), 1 h prior to feeding (−1 h), upon commencement of feeding (0 h), 1 h after feeding (+1 h) and 3 h after feeding (+3 h). Two unfed groups were sampled at +1 h, and +3 h and served as the unfed group. Total RNA extraction, cDNA synthesis, and qRT-PCR were conducted as described above. The NUCB2 mRNA is expressed as a percentage of the normalized NUCB2 expression at −3 h.

### Food Deprivation Induced Changes in NUCB2 mRNA Expression in Goldfish Hypothalamus and Liver

The homeostatic alterations induced by food deprivation can change the expression of appetite regulatory peptides within the neuroendocrine system [25]. This study examined the changes in NUCB2 mRNA expression in the hypothalamus and liver of goldfish during food deprivation over a seven-day period. Five groups of weight-matched goldfish (*n = 6/group*) were acclimated to tank conditions and fed daily at a scheduled time for 2 weeks. Two groups of fish were then not fed or exposed to food for 3 or 7 days, while the two control groups were fed daily. Hypothalamus and liver were sampled on days 3 and 7 from the fasted fish, and from the fed fish at 3 h post-feeding. After a 7 day fast, one additional unfed group was re-fed and sampled at 3 h post-re-feeding. Tissue extraction and relative gene expression quantification were conducted as described previously.

### Effects of the Feeding Status on Serum Nesfatin-1 Levels in Goldfish

If nesfatin-1 indeed is an endogenous anorexigen, we expect to see an association between the nutritional status of the animal and changes in circulating nesfatin-1 levels. To investigate meal related changes in nesfatin-1 levels, serum samples were collected from *ad libitum* fed, 1 and 3 hour post-fed, as well as 24 h fasted goldfish (n = 5/group). Blood samples were collected from the caudal vein and serum was collected immediately by centrifugation (7000 rpm for 9 minutes at 4 C) and stored at −20 C. Serum nesfatin-1 levels were measured using the Nesfatin-1 (1–82) (Rat) ELISA kit (Cat. No. EK-003-2, Phoenix Pharmaceuticals Inc., CA). This assay was validated for use in fish and the quality controls provided with the kit were within the expected range. To confirm the efficiency of the ELISA with synthetic goldfish nesfatin-1, a standard curve was plotted from the absorbance and logarithmic values for the different amounts of peptide. The amount of immunoreactive material was determined using a non-linear regression curve-fit, which was used to quantify the concentration of nesfatin-1 in serum samples.

### Western Blot Analysis

Brain samples were harvested from goldfish and Fischer 344 rats (for positive control) and sonicated for 30 seconds on ice in Lysis Buffer (135 mM NaCl, 1 mM MgCl, 2.7 mM KCl, 20 mM Tris buffer pH 8, 1% Triton-X, 10% Glycerol, 0.5 mM Sodium vanadate, 0.2 mM phenylmethanesulfonylfluoride, 10 mM NaF). The whole tissue protein lysates were centrifuged at 12,000 rpm for 20 minutes at 4 C to remove cellular debris. The supernatant was used as the soluble protein extract. Final protein concentrations were determined by Bio-Rad Protein Assay (Bio-Rad, Hercules, CA). Gel samples were prepared by mixing the protein samples with gel loading buffer (4% SDS, 0.05% bromophenol blue (w/v), 20% glycerol, 1% mercaptoethanol (v/v) in 0.1 M Tris buffer, pH 6.8). Samples were incubated at 100 C for 5 minutes. They were subsequently loaded on a 10% SDS-polyacrylamide gel. After SDS-PAGE, proteins were transferred to a nitrocellulose membrane (Pall, Mexico) for 2 h at 4 C. The membrane was blocked in Tris-buffered saline [TBS; 10 mM Tris, 150 mM NaCl, 0.05% Tween, v/v] containing 5% (w/v) nonfat milk (Carnation, Nestlé, Glendale, CA). After 1 h, the membranes were incubated in primary antibody (polyclonal anti-nesfatin-1, Phoenix Pharmaceuticals, Burlingame, CA) diluted 1∶1000 in TBS containing 5% (w/v) non-fat milk overnight. Membranes were later washed three times with TBS containing 0.5% non-fat milk. Secondary antibody (anti-rabbit IgG HRP-linked, New England Biolabs, Pickering, Canada) diluted 1∶40,000 in TBS was added to the membrane. The membrane was subsequently washed twice in TBS containing 0.5% non-fat milk then twice using TBS only. Ultimately, the membrane was developed using ECL Western Blot Substrate (Pierce, Rockford, IL) as per manufacturer's instructions.

### Nesfatin-1-like ir in the Brain and Gut

Goldfish brain and J-loop region (anatomical equivalent of the mammalian stomach) were dissected under saline and immediately transferred to 4% paraformaldehyde for a 3 h incubation period (4 C) (n = 6 fish). After fixation, tissues were washed with 70% ethanol, embedded and sectioned sagitally (5 µm thickness). Sections were deparaffinized with xylene (3×10 minutes, 25 C) and rehydrated in a graded ethanol series. DAKO® serum-free protein block reagent (DAKO Corporation, CA) was used to block the section for 10 minutes prior to incubation with rabbit anti-nesfatin-1 primary antibody (Pheonix Pharmaceuticals; 1∶500 dilution) for 18 h at 4 C. After incubation, slides were washed with 1X PBS (3×10 minutes, 25 C) and incubated with goat anti-rabbit Texas Red® IgG (Red-Nesfatin-1; Vector Laboratories, CA; 1∶1000 dilution) secondary antibody for 1 h at room temperature. Lastly, slides were washed with 1X PBS (3×10 minutes, 25°C) and mounted with Vectashield® mounting medium containing DAPI (Blue nuclear stain; Vector Laboratories). Negative controls were treated with secondary antibody only. All images were taken using a Nikon Eclipse Ti-inverted fluorescence microscope (Nikon Canada, Mississauga, Canada) connected to a Dell HP Workstation computer and NIS-elements basic research imaging software (Nikon Canada, Mississauga, Canada). Representative images were chosen from a large number of sections assessed from tissues of all six fish. Anatomical localization and nomenclature were based on the goldfish brain atlas [26].

### Effects of IP Injections of Nesfatin-1 on Food Intake of Goldfish

To determine the influence of nesfatin-1 on food intake in goldfish, *gf*nesfatin-1 was peripherally administered. Seven groups of six, weight-matched goldfish (Body Weight [BW]  = 36.61±7.20 g) were IP injected with 500, 50, 5. 0.5 and 0 ng/g BW *gf*nesfatin-1 (both saline and 50 ng/g BW groups were repeated IP injections because these studies were conducted on separate days). Goldfish were allowed to acclimate to tanks over a period of two weeks prior to the study to reduce the effects of stress. The fish were fed at a scheduled feeding time (12:00 h) once a day with 4% BW ration per fish and food intake was measured daily for 1 h post-feeding. On the day of the study, goldfish were anesthetized prior to their daily scheduled feeding time and treated with an injection of either 100 µl saline alone or peptide using a 1 ml syringe and 25^(5/8)^ G needle (BD, Oakville, Canada). Subsequently, fish were returned to aquariums and fed at 5 minutes post-injection, at which all fish were active and swimming. Uneaten food pellets were removed 1 h after feeding, dried for 24 h, and weighed the next day. Quantification of food intake was determined by subtracting the dry weight of the total amount of food given by the dry weight of the food removed from the aquarium after 1 h. All remaining studies were conducted with the same acclimation and feeding protocols unless otherwise stated.

### Effects of ICV Injections of Nesfatin-1 on Food Intake of Goldfish

The anorectic effect of nesfatin-1 was discovered mainly after directly administering it into the brain of rodents [1]. We used ICV injections, a very well characterized technique in goldfish to test the central effects of nesfatin-1 on feeding. ICV injections were conducted as previously described [27]. Briefly, three groups of six weight-matched goldfish (BW  = 33.71±9.71 g) were used for central injections of 25, 0.5 and 0 ng/g BW *gf*nesfatin-1. Subsequently, fish were returned to their respective aquariums and fed 6% BW ration per fish following a five minute recovery period. Uneaten food pellets were removed after 1 h and measured as previously described.

### Statistical analysis

Statistical analyses for qRT-PCR, food intake studies and nesfatin-1 ELISA data were conducted using a one-way ANOVA followed by Student-Newman-Keuls multiple comparison test (GraphPad Prism 4, San Diego, CA). Significance was considered at P<0.05. Data is expressed as mean ± SEM.

## Results

### Identification of Teleost NUCB2 Genes and Sequencing of the Goldfish NUCB2 mRNA

**Figure 1:**
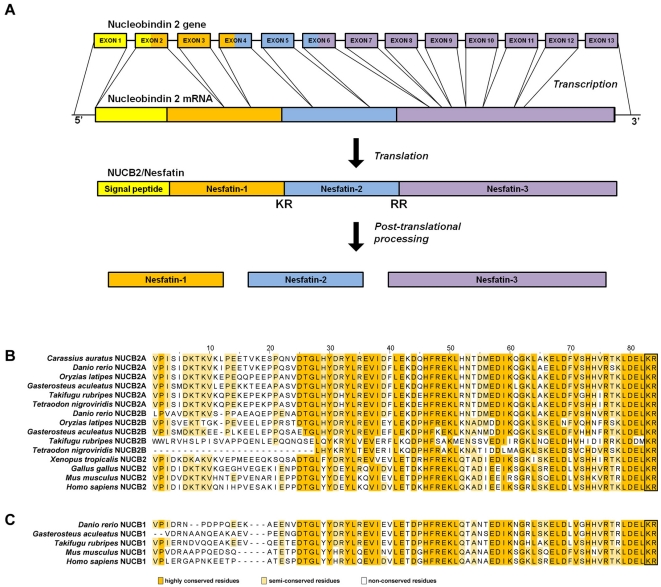
A figure showing the putative processing of NUCB2 and comparison of nesfatin-1 sequences. Schematic outline of zebrafish NUCB2 gene and the formation of nesfatin-1 (**A**). In this figure, the boxes which make up the NUCB2 gene represent exons 1–13 and interspaced lines represent introns 1–12. The proposed scheme for NUCB2 prepropeptide and putative cleavage sites from the NUCB2 gene are shown. All other teleosts examined have similar, highly conserved intron:exon organization for the NUCB2A gene, except for pufferfish (*Tetraodon nigroviridis*) and fugu (*Takifugu rubripes*) which have exon 1 deleted. **Figure 1B** shows the amino acid sequence alignment of the nesfatin-1 region of NUCB2, NUCB2A and NUCB2B (teleost only). **Figure 1C** is the alignment of NUCB1 from cartilaginous fish, bony fish, amphibians, birds and mammals. The name of the species is given on the left side of the alignment and the number of amino acids in the peptide is provided above the alignment. The colored amino acids highlight the differences in conservation of the amino acids between species within the nesfatin-1 region of NUCB2 and NUCB1. In addition, the putative cleavage sites are shown in the box. Species names used in the alignment were as follows: zebrafish (*Danio rerio*), stickleback (*Gasterosteus aculeatus*), medaka (*Oryzias latipes*), green pufferfish (*Tetraodon nigroviridis*), fugu (*Takifugu rubripes*), Western clawed frog (*Xenopus tropicalis*), chicken (*Gallus gallus*), mouse (*Mus musculus*), and human (*Homo sapiens*).

The NUCB2 gene structure is remarkably well conserved among both teleosts and other vertebrates examined. NUCB2 in the cyprinids, *Carassius auratus* (goldfish) and *Danio rerio* (zebrafish) most closely aligned together (**Figure 1**). NUCB2A and NUCB2B gene sequences in zebrafish consist of 13 exons interspaced by 12 introns (**Figure 1A**). The same gene organization is found in *Oryzias latipes* (medaka) and *Gasterosteus aculeatus* (stickleback). NUCB2A in *Takifugu rubripes* (fugu) and *Tetraodon nigroviridis* (green pufferfish) both have 12 exons interspaced by 11 introns and this appears to be due to the deletion of exon 1. (**Figure 1A**). Goldfish NUCB2 mRNA was amplified from the brain and sequenced (**Figure S1**; GenBank accession number: HM065567). The deduced NUCB2 protein is composed of 499 amino acid residues, with a 23 amino acid signal region and a 476 amino acid protein (**Figure S1**). In addition, the proposed prohormone convertase cleavage site (Lys-Arg) is ubiquitously conserved (**Figure 1B**). The nesfatin-1 region in the goldfish NUCB2A sequence has high percent similarity to the nesfatin-1 region of NUCB2 or NUCB2A (teleost only) of other species including: zebrafish (*Danio rerio*; 94%), medaka (*Oryzias latipes*; 91%), stickleback (*Gasterosteus aculeatus*; 86%), green pufferfish (*Tetraodon nigroviridis*; 84%), fugu (*Takifugu rubripes*; 83%), Western clawed frog (*Xenopus tropicalis*; 64%), chicken (*Gallus gallus*; 66%) mouse (*Mus musculus*; 57%), and human (*Homo sapiens*; 63%) (**Figure 1B**). The nesfatin-1 region of the NUCB1 sequences share the lowest similarity to NUCB2 in the N-terminal of the peptide (**Figure 1C**). Interestingly, much of the C-terminal region of the putative nesfatin-1 sequence are highly conserved including the proposed prohormone convertase cleavage site. To date, there have been no reports on the processing of nesfatin-1 from NUCB1 or any reports of the involvement of NUCB1 in food intake or glucose homeostasis.

Sequences were obtained from the Ensembl database and GenBank and were aligned using ClustalW. Ensembl database accession numbers of the sequences were as follows; *Gasterosteus aculeatus* (NUCB1, ENSGACP00000016912; NUCB2A, ENSGACP00000020611; NUCB2B, ENSGACP00000008999), *Oryzias latipes* (NUCB2A, ENSORLP00000008372; NUCB2B, ENSORLP00000001612), *Tetraodon nigroviridis* (NUCB2A, ENSTNIP00000012254; NUCB2B, ENSTNIP00000005671), *Takifugu rubripes* (NUCB1, ENSTRUP00000000823; NUCB2A, ENSTRUP00000044315; NUCB2B, ENSTRUP00000038477). GenBank accession numbers of the sequences were as follows; *Carassius auratus* (NUCB2A, HM065567.1), *Danio rerio* (NUCB1, NM_001045463.1; NUCB2A, NM_201493.1; NUCB2B, NM_201479.1), *Xenopus tropicalis*, (NUCB2, AAH90107.1), *Gallus gallus* (NUCB2, NP_001006468.1), *Mus musculus* (NUCB1, AAH72554.1; NUCB2, AAH10459.1) and *Homo sapiens* (NUCB1, NP_006175.2; NUCB2, NP_005004.1).

**Figure 2:**
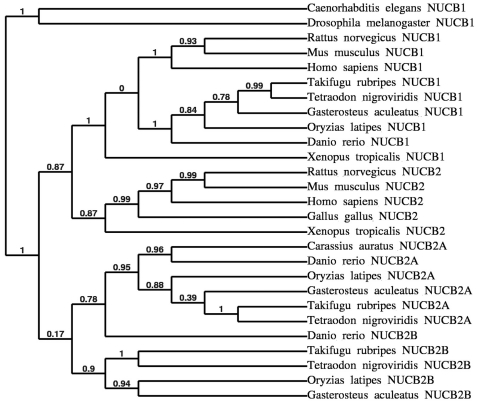
Phylogenetic analysis of nucleobindin gene sequences. NUCB2A and NUCB2B (teleost only), and NUCB1 amino acid sequences. Each node has a bootstrap value which was obtained for 500 replicates. GenBank Accession Numbers or Ensembl identification numbers are as follows; *Caenorhabditis elegans* (NUCB1, NM_171763.3), *Drosophila melanogaster* (NUCB1, AAF49304.3), *Rattus norvegicus* (NUCB1, AAI00644.1), *Mus musculus* (NUCB1, AAH72554.1; NUCB2, AAH10459.1), *Homo sapiens* (NUCB1, NP_006175.2; NUCB2, NP_005004.1), *Takifugu rubripes* (NUCB1, ENSTRUP00000000823; NUCB2A, ENSTRUP00000044315; NUCB2B, ENSTRUP00000038477), *Tetraodon nigroviridis* (NUCB2A, ENSTNIP00000012254; NUCB2B, ENSTNIP00000005671), *Gasterosteus aculeatus* (NUCB1, ENSGACP00000016912; NUCB2A, ENSGACP00000020611; NUCB2B, ENSGACP00000008999), *Oryzias latipes* (NUCB2A, ENSORLP00000008372; NUCB2B, ENSORLP00000001612), *Danio rerio* (NUCB1, NM_001045463.1; NUCB2A, NM_201493.1; NUCB2B, NM_201479.1), *Xenopus tropicalis*, (NUCB2, AAH90107.1), *Gallus gallus* (NUCB2, NP_001006468.1), and *Carassius auratus* (NUCB2A, HM065567.1).

In the phylogenetic analysis (**Figure 2**), the amino acid sequence of goldfish NUCB2A was clustered with the NUCB2A sequences of other teleostean fishes, while most strongly clustering with zebrafish NUCB2A. In addition, the teleostean NUCB2B sequences were divided from the NUCB2A sequences, although zebrafish NUCB2B is more closely grouped with NUCB2A than with other NUCB2B sequences. NUCB1, NUCB2, and the teleostean NUCB2 paralogous were all divided from mammalian, amphibian, avian and invertebrate homologues with high bootstrap value.

### Tissue Distribution of NUCB2 mRNA in Goldfish

**Figure 3:**
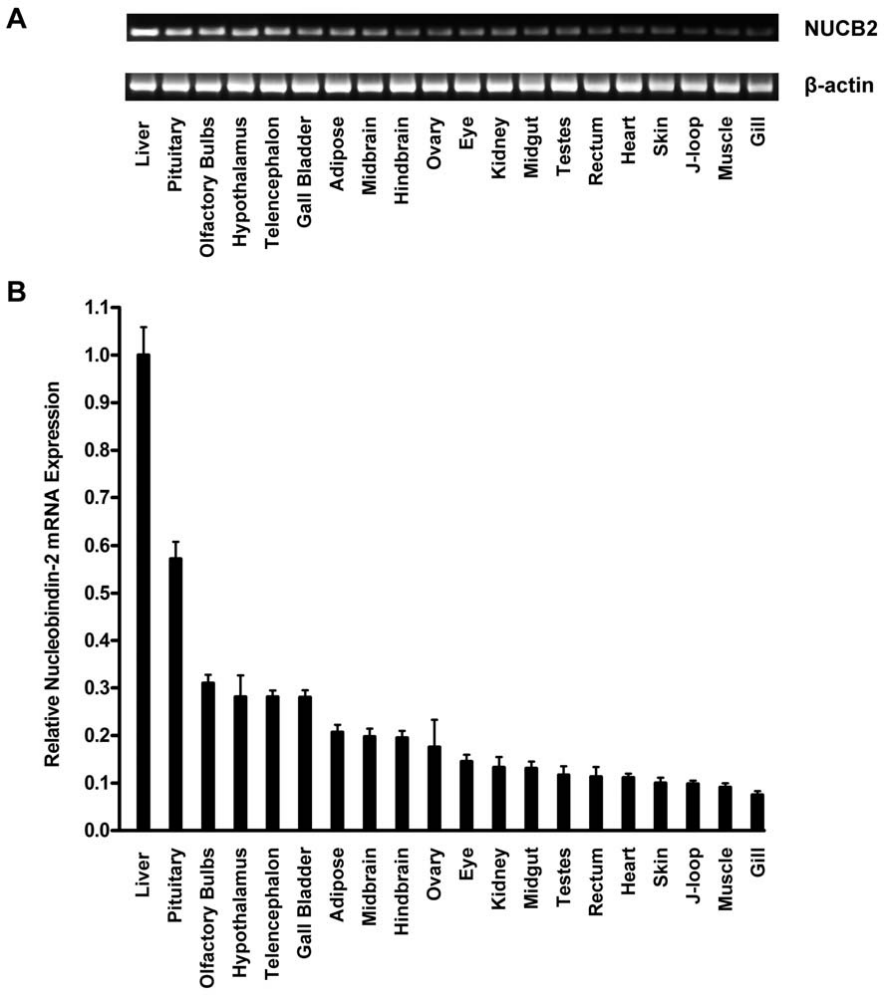
Differential expression of NUCB2 mRNA in goldfish tissues. NUCB2 and beta-actin cDNA amplicon products from the tissues of *Carassius auratus* by reverse transcriptase PCR (**A**). More precise quantitative data was obtained by using Real-Time Quantitative Reverse Transcription PCR (**B**). The results were normalized to β-actin, which served as a control to verify the amount and quality of samples (*n = 5 fish*).

In goldfish, reverse transcriptase PCR detected abundant NUCB2 mRNA expression in the liver and pituitary in addition to various regions of the brain, including the olfactory bulbs, hypothalamus, telencephalon, midbrain and hindbrain (**Figure 3A**). More quantitative measurements were made by qRT-PCR. Relative to the liver, lower levels of NUCB2 mRNA expression was detected in the adipose tissue, ovary, eye, kidney, and midgut (**Figure 3B**). While the lowest levels of NUCB2 mRNA expression were found in the muscle and gill (**Figure 3B**).

### Periprandial Expression of NUCB2 mRNA in the Goldfish Hypothalamus and Liver

**Figure 4:**
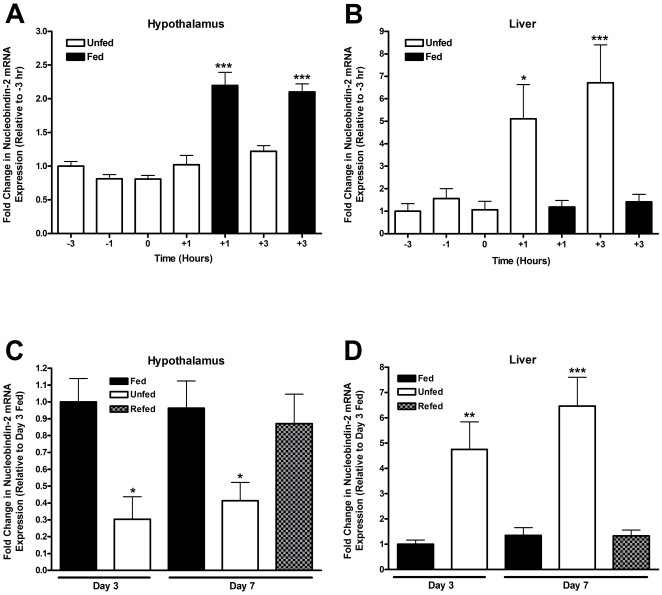
Feeding status affects the expression of NUCB2 mRNA within the hypothalamus and liver of goldfish. Pre- and post-prandial changes in the expression of NUCB2 mRNA in the (**A**) hypothalamus and (**B**) liver of goldfish. The mRNA expression of nesfatin was normalized to β-actin and represented relative to the −3 hour scheduled feeding time group. Asterisks represent significant differences between groups at the same time point. Food deprivation decreased expression of NUCB2 mRNA in the goldfish hypothalamus (**C**) and increased its expression in the liver (**D**). Three and 7 day NUCB2 mRNA expression is represented as the normalized percentage of the NUCB2 mRNA expression in the 3 day food-deprived fish. One-way ANOVA, Newman-Keuls Multiple Comparison Test. Asterisks represent significant differences between groups at the same time point. * P<0.05, ** P<0.01, *** P<0.001. (n = 6 fish/group).

There was an ∼2-fold increase in NUCB2 mRNA expression in the hypothalamus of fed goldfish at 1 and 3 h post-feeding compared to unfed controls (P<0.001, one-way ANOVA) (**Figure 4A**). Interestingly, there was an ∼5 and 6-fold increase in the levels of NUCB2 mRNA expression in the liver of unfed animals at 1 and 3 h post-feeding compared to fed controls, respectively (P<0.05, P<0.001) (**Figure 4B**). No changes in NUCB2 mRNA were detected in unfed groups before the scheduled feeding time and in fed animals after the scheduled feeding time (**Figure 4B**). A single melting peak was detected by melting temperature analyses for both β-actin and NUCB2 mRNA indicative of the specificity of the reaction (data not shown).

### Food Deprivation Induced Changes in NUCB2 mRNA Expression in Goldfish Hypothalamus and Liver

NUCB2 mRNA expression in the hypothalamus of goldfish fed daily was ∼3 and 2-fold higher compared to fish deprived of food for 3 or 7 days, respectively (P<0.05, one-way ANOVA) (**Figure 4C**). When 7 day food-deprived fish were re-fed, NUCB2 mRNA expression in the hypothalamus increased 2-fold above that of the 7 day fasted fish (P<0.05, one-way ANOVA) (**Figure 4C**). Similar to the unfed fish in the periprandial study, NUCB2 mRNA expression in the liver was significantly elevated in food-deprived fish (**Figure 4D**). A 4.8-fold and 6.5-fold increase in NUCB2 mRNA expression was detected in the liver of fish that were food deprived for 3 and 7 days, respectively. Re-feeding after a 7-day food-deprivation brought the NUCB2 mRNA to control levels 3 h post-feeding. No significant changes in NUCB2 mRNA levels were observed between 3 and 7 day food-deprived groups. All groups are expressed relative to the level of NUCB2 mRNA expression of the 3 day fed group (**Figure 4C–D**).

### Effects of Feeding Status on Serum Nesfatin-1 Levels

**Figure 5:**
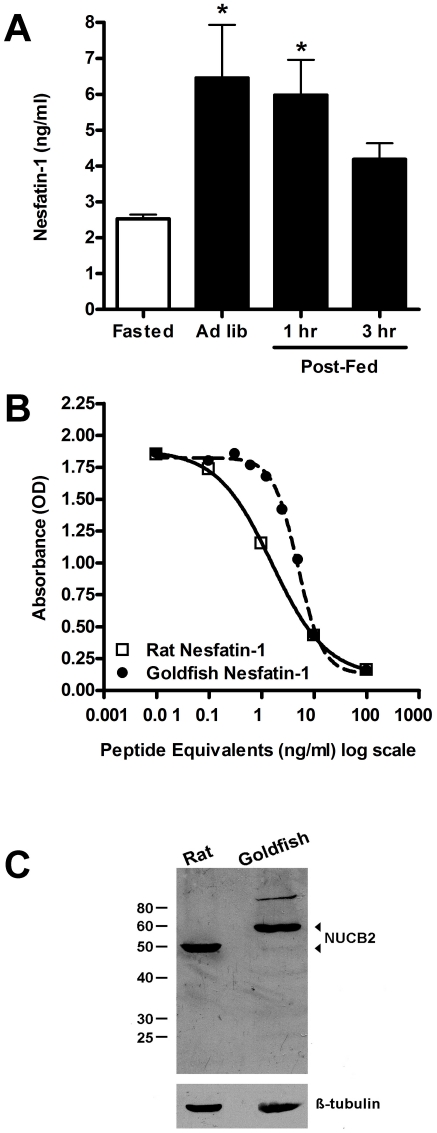
One-day food deprivation reduces serum nesfatin-1 levels in goldfish. Elevated serum nesfatin-1 levels can be detected up to 1 hour post-feeding but is reduced 3 hours post-feeding (**A**). Nesfatin-1 ELISA standard curve shows strong cross reactivity with goldfish nesfatin-1 over a range of 1–100 ng/ml (**B**). Western blot analysis confirmed the specificity of polyclonal pronesfatin antibody (**C**). Rodent and goldfish pronesfatin (NUCB2) were detected at their predicted molecular weights, ∼50 and ∼59 kDa, respectively, β-tubulin was added as a loading control and detected in both rat and goldfish tissues. No bands representing processed nesfatin-1 (∼9.5 kDa) was found in both rat and goldfish brain.

Food-deprived goldfish had significantly less nesfatin-1 in serum than regularly fed controls with 2.52±0.26 and 6.45±3.31 ng/ml, respectively (P<0.05) (**Figure 5A**). In addition, serum nesfatin-1 levels remained significantly elevated in circulation (5.97±2.19 ng/ml) up to 1 hr after feeding (P<0.05) (**Figure 5A**). A standard curve was plotted from premeasured amounts of the synthetic *gf*nesfatin-1 and the amount of immunoreactive material was calculated from a non-linear regression curve-fit of the standard curve (**Figure 5B**). The ELISA for goldfish nesfatin-1 is sensitive over a range of 1–20 ng/ml. The EC_50_ for the goldfish nesfatin-1 standard curve was found to be approximately 4.9 ng/ml. It is concluded from the standard curve that the antiserum is at least 1-order of magnitude more sensitive to rat nesfatin-1 than to the goldfish nesfatin-1 peptide (**Figure 5B**). In order to confirm specificity of the anti-nesfatin-1 antibody, which probes nesfatin-1 and the precursor NUCB2, a western blot of goldfish whole brain tissue lysates was conducted. Due to the differences in amino acids constituting the peptides, rat NUCB2 is expected to be ∼50 kDa, while the goldfish NUCB2 is ∼59 kDa. As expected, the antibody targeted full-length NUCB2 in rat and goldfish brain samples (**Figure 5C**). No band representing the processed nesfatin-1 (expected size: ∼9.5 kDa) was detected in either rat or goldfish brain samples.

### Nesfatin-1-like ir in the Hypothalamus

**Figure 6:**
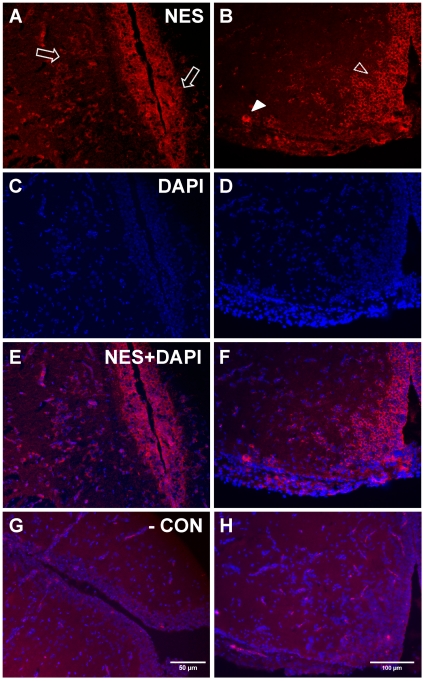
Characterization of nesfatin-1 like immunoreactivity within the goldfish hypothalamus. Immunohistochemical staining of goldfish hypothalamus for nesfatin-1-like immuunoreactivity (**A**, **B**; red), DAPI (**C**, **D**; nuclei; blue) and the merged image of nesfatin-1 and DAPI is shown in **E** and **F**. Representative cells that are immunopositive for nesfatin-1 in the NAT (A; open arrows) or in the NLTp (B; solid arrow) or in the NLTl (B; solid arrowhead) are shown. Figures **G** and **H** represents a no-primary antibody negative control, which was labeled only with secondary antibodies. Representative images were taken of 30 sections from 6 separate goldfish hypothalami.

A sagittal view of the goldfish brain stained with nesfatin-1 antiserum, revealed a large number of ir cell bodies within the goldfish hypothalamus (**Figure 6A–F**). A relatively high proportion of these cell bodies were found within the nucleus anterior tuberis (NAT) (**Figure 6A, E**). More caudally, many nesfatin-1-like ir neurons were observed within the nucleus lateralis tuberis (NLT). These fluorescent perikarya were found in the central (NLT*p*) and lateral regions (NLT*l*) of the NLT (**Figure 6B, F**). All immunoreactive cells within the brain were stained clearly with the nuclear stain DAPI (**Figure 6C, D**). Immunohistochemical analysis showed strong cytoplasmic staining and no nesfatin-1-like ir was observed in nerve fibers. No staining was observed in brain sections where primary antibody was omitted (**Figure 6G, H**). In order to confirm specificity of the anti-nesfatin-1 antibody, which probes nesfatin-1 and the precursor NUCB2, a western blot of goldfish whole brain tissue lysates was conducted.

### Nesfatin-1-like ir in the J-loop

**Figure 7:**
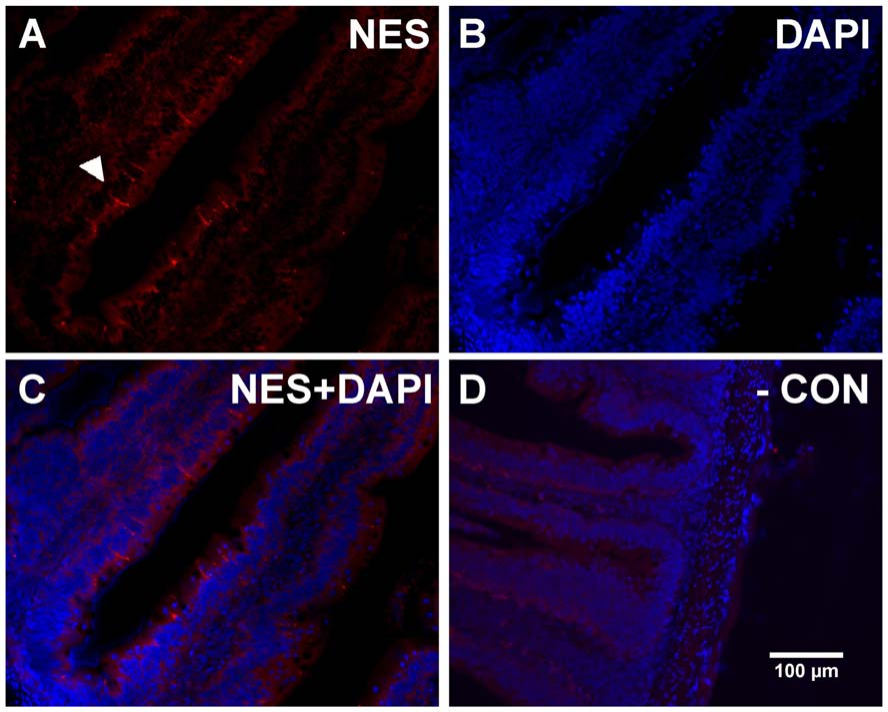
Nesfatin-1 like immunoreactivity is also present within the goldfish gut. Nesfatin-1-like immunoreactivity found within enteroendocrine cells of the goldfish gut. Immunohistochemical localization shows nesfatin-1-like immunoreactive cells (arrows) in the intestinal villi of the gut (**A**). The nuclear stain DAPI (**B**) and merged images (**C**) show nesfatin-1-like immunoreactive cells along the base of the intestinal villi (solid arrow). The inset in **C** shows a representative nesfatin-1-like immunoreactive cell under high magnification (100x). A negative control where no primary, but only secondary antibody showed no staining (**D**). Representative images were taken of 18 sections from 6 separate goldfish guts.

Longitudinal sections of the goldfish anterior intestine stained for nesfatin-1-like ir within enteroendocrine like cells of the intestinal villi (**Figure 7A**). Most of these cells were scattered deep within the folds of the villi, however, some of the cells were dispersed along the apical regions of the villi. Lightly stained blebbed processes emanating from the enteroendocrine cells are visible and appear to project towards the lumen (**Figure 7A, C**). The nuclear stain, DAPI, stained clearly within the cell bodies of nesfatin-1-like ir cells and non-ir cells (**Figure 7B, C**). No staining was observed in sections in which only secondary antibody was used (**Figure 7D**).

### Effects of IP Injections of Nesfatin-1 on Food Intake of Goldfish

**Figure 8:**
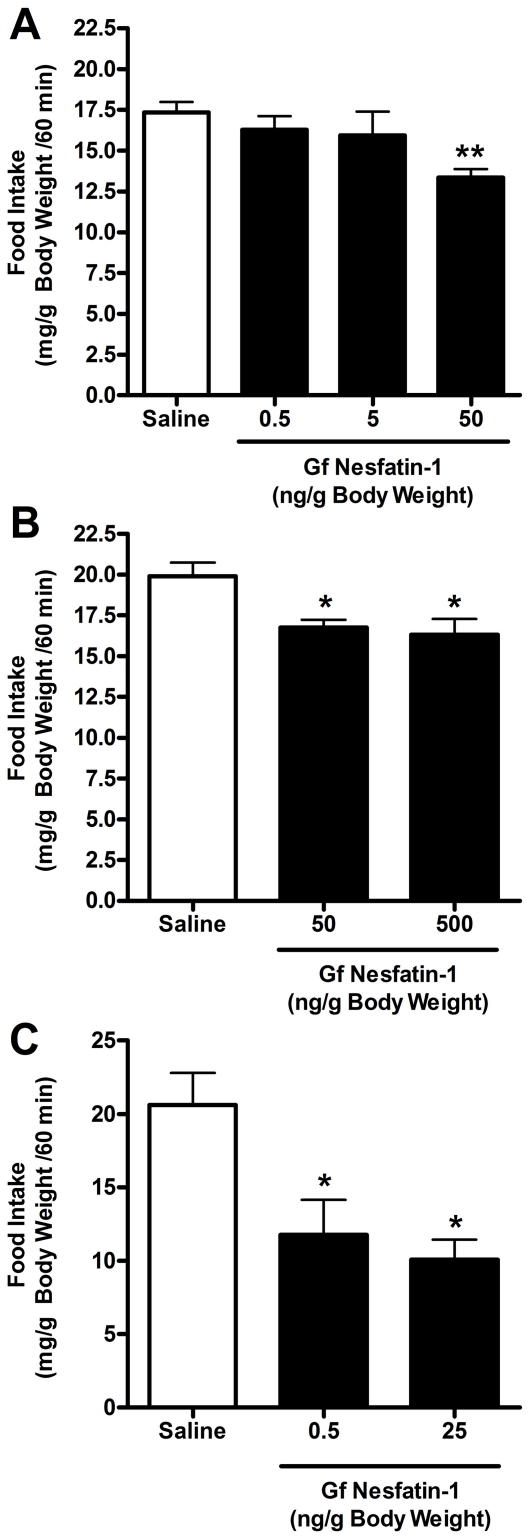
Effects of goldfish (*gf*) nesfatin-1 administration on food intake over 1 h post-injection. Effects of intraperitoneal (IP) injections of relatively low doses of *gf*nesfatin-1 (0.5, 5, and 50 ng/g body weight [BW]) (**A**). Effects of IP injections of relatively high doses of *gf*nesfatin-1 (50, and 500 ng/g BW) (**B**). Effects of intracerebroventricular (ICV) injections of *gf*nesfatin-1 on food intake over 1 hour (**C**). A 0.5 and 25 ng/g BW ICV injection of the *gf*nesfatin-1 significantly reduced food intake over one hour by ∼43 and 50%, respectively. One-way ANOVA, Newman-Keuls Multiple Comparison Test. ** P<0.01, * P<0.05. (*n = 6 fish/group*).

A single IP injection of 50 ng/g BW (5.3 pmol/g BW) *gf*nesfatin-1 reduced food intake maximally by 23% compared to saline treated fish (P<0.01) (**Figure 8A, B**). Additionally, an injection of 500 ng/g BW (53 pmol/g BW) *gf*nesfatin-1 produced similar reductions in food intake with an 18% reduction compared to controls (**Figure 8B**) (P<0.05). Furthermore, lower doses (0.5 and 5 ng/g BW, 0.053 and 0.53 pmol/g BW, respectively) of *gf*nesfatin-1 were ineffective in altering food intake during the study period (**Figure 8A**).

### Effects of ICV Injections of Nesfatin-1 on Food Intake of Goldfish

ICV injections of synthetic *gf*nesfatin-1 produced enhanced reductions in food intake. An ICV injection of 0.5 or 25 ng/g BW (0.053 or 2.65 pmol/g BW, respectively) of *gf*nesfatin-1 reduced food intake by 43, and 51%, respectively (P<0.05, one-way ANOVA) (**Figure 8C**).

## Discussion

We employed a multifaceted approach to characterize the structure, distribution, and appetite regulatory effects of nesfatin-1 in non-mammalian vertebrates for the first time. The goldfish NUCB2A mRNA sequence reported here is 1568 bp and encodes a 23 amino acid signal peptide followed by a 476 amino acid NUCB2A prohormone. Comparative analysis of the sequence we obtained indicates that goldfish NUCB2 sequence belongs to a subgroup of NUCB2 sequences in teleost fishes called NUCB2A. The presence of duplicated NUCB2 genes in teleost fishes presumably arose through an independent tetraploidization known as 3R (third round of genome duplication) [28], [29]. A wider phylogenetic analysis of NUCB2 sequences in several teleosts shows strong clustering for 2 paralogous NUCB2 genes (NUCB2A and NUCB2B). In addition, the proposed prohormone convertase cleavage site (Lys-Arg) is ubiquitously conserved in both paralogous NUCB2 genes, suggestive that the putative nesfatin-1 peptide can be cleaved from the larger NUCB2 precursor. The complexity of the goldfish genome suggests that an additional NUCB2B gene may exist. Furthermore, the duplication of the NUCB2 gene suggests a potential for redundancy and subspecialization possibly contributing to a more complicated nesfatin-1 system in fishes than in mammals. Interestingly, NUCB1 in teleosts is also highly conserved and retains the proposed prohormone convertase cleavage site. However, to date no evidence exists for NUCB1 in the regulation of food intake and energy expenditure.

Oh-I et al. (2006) initially proposed the post-translational processing of NUCB2 through specific cleavage sites by prohormone convertases (PCs). These cleavage sites which consist of the basic amino acid pairs, Lys-Arg and/or Arg-Arg remain conserved in goldfish. It is well known that PC1/3 and PC2 are the major PCs involved in prohormone processing. In addition, previous studies have described the presence of PC1/3 and PC2 in early evolution [18] including in fish [19], [20]. Thus, the prohormone convertase processing of NUCB2 is expected to yield nesfatin-1, nesfatin-2, and nesfatin-3, similar to what has been proposed in mammals. However, in our Western blot analyses, we did not see the expected 9.5 kDa band representing nesfatin-1 in goldfish or rat brain. The commercially available antibody used in this study is the most widely reported in the nesfatin-1 literature. To date, none of these reports were able to show endogenous, fully processed nesfatin-1 using the Western blot technique in any species. It is possible that processed nesfatin-1 levels are below the detectable range. In addition, previous studies have speculated that NUCB2 is only processed upon secretion into the circulation to generate the 9.5 kDa peptide [9]. Given that the antibody used here detects the precursor form of the peptide, the immunoreactivity we report represents the precursor as well as the processed peptides. In goldfish, the predicted nesfatin-1 and 2 are highly conserved and are 82 and 81 amino acids long, respectively. Nesfatin-3 is longer in goldfish (309 amino acids) than in rat (231 amino acids). In addition, the mid-segment of nesfatin-1 is well conserved among the vertebrates examined here. According to Shimizu et al., (2009) the mid-segment of nesfatin-1 (24–53) is the bioactive appetite inhibitory segment. The identification of a highly conserved NUCB2 and nesfatin-1 in lower vertebrates lays the foundation for further studies on this novel hormone.

In rats, NUCB2 is found in many regions of the brain, including the paraventricular nucleus (PVN), arcuate nucleus (ARC), supraoptic nucleus (SON), and in the lateral hypothalamic area (LHA), and the zona incerta [1]. In addition, nesfatin-1-ir has been detected in the nucleus of the solitary tract (NTS) and dorsal nucleus of the vagus nerve [1], [2]. Furthermore, there is increasing evidence for the presence of nesfatin-1 in peripheral tissues. Pronesfatin is found in the oxyntic cells of the fundic mucosa [9] and in the beta cells of the Islets of Langerhans [10], [11]. The current study extends these findings by describing the tissue distribution of nesfatin-1 within a non-mammalian vertebrate species. Goldfish NUCB2 is expressed in various tissues. The highest expression was found in the liver, pituitary, olfactory bulbs, hypothalamus, telencephalon, gall bladder, and adipose tissue of goldfish. Providing further support, our immunohistochemical studies show the presence of nesfatin-1-like ir within the hypothalamus and preoptic areas of the goldfish brain. Nesfatin-1-like ir was detected in enteroendocrine like cells lining the J-loop, a structure homologous to the mammalian stomach. These observations expand the mRNA expression studies by confirming the presence of nesfatin-1-like ir within the anterior intestine. These results from a distantly related lower vertebrate highlights the growing evidence in support of a role for nesfatin-1 as a novel brain-gut regulatory peptide.

The nucleus lateralis tuberis (NLT) of goldfish is considered as a teleostean homologue to the mammalian arcuate nucleus [30], [31]. The largest number of nesfatin-1-like ir cell bodies were found in the NLT of goldfish. Interestingly, the NLT neurons which stain for nesfatin-1-like ir are also known to innervate the goldfish pituitary [32], [33]. Unlike in tetrapods, which release neurohormones through the classical hypothalamic-pituitary portal system, teleost fish have processes that originate from the neurohypophysis and project through the adenohypophysis towards the anterior and intermediate lobes [34]. Furthermore, the abundance of NUCB2 observed in the pituitary is suggestive of a role for nesfatin-1 in the regulation of the hypothalamo-pituitary axis. To date, many other well established neuropeptides with mammalian orthologues have been characterized within the NLT of the goldfish brain. Immunoreactive NPY and AgRP neurons as well as *in situ* hybridized preprocholecystokinin and preprogalanin perikarya have been described in the NLT [35]–[37]. In addition, cell bodies for appetite-suppressing POMC and the melanocortin 4 receptor (MC-4R) ir cell bodies have been described within the NLT [38]. The localization of nesfatin-1-like ir neurons in the NLT suggest possible interactions of nesfatin-1 with other peptides in regulating food intake. Current research in mammals suggests that nesfatin-1 may act through a central oxytocin system originating from the oxytocin neurons in the PVN which exert influence on POMC neurons within the NTS [15], [39]. Additionally, nesfatin-1 may act either in series or independently through the central melanocortin system by directly acting upon POMC neurons in the ARC to affect oxytocin neurons in the paraventricular nucleus (PVN) [16]. Whether or not the central physiological effects of nesfatin-1 are mediated in a similar manner as suggested by the distribution pattern in the goldfish hypothalamus remains to be determined.

NUCB2 expression has been shown to selectively decrease in the PVN under starved conditions, while re-feeding returned NUCB2 back to control levels [1]. Our findings confirm and extend these findings in non-mammalian vertebrates. Goldfish NUCB2 mRNA levels remained statistically indistinguishable in the hypothalamus until after feeding. At 1 hour and 3 hours post-feeding, the levels of NUCB2 increased by ∼2-fold compared to unfed controls. In addition, an ∼3 and 2-fold difference in NUCB2 mRNA was observed in the hypothalamus of fish deprived of food for 3 or 7 days, respectively. Furthermore, a 2-fold increase was detected in the hypothalamus of animals re-fed after a 7 day food deprivation, compared to *ad libitum* fed animals. Previous studies in rats have shown that circulating levels of nesfatin-1 decrease during fasting and increase significantly after a meal [15]. In addition, Stengel et al., (2009) observed a down-regulation of NUCB2 mRNA in gastric endocrine cells during fasting and increases in NUCB2 mRNA in the stomach and brain of fed rodents. Since the changes observed in NUCB2 mRNA are not indicative of the synthesis or release of the peptide, we tested goldfish blood serum during different nutritional challenges to examine the levels of nesfatin-1 in circulation. In accordance with our mRNA expression data we also observed significant decreases in the levels of serum nesfatin-1 in 24 h food-deprived goldfish compared to *ad libitum* fed controls. Interestingly, NUCB2 mRNA in the liver was elevated during food deprivation. During food deprivation, liver gluconeogenesis acts as a primary source of glucose to facilitate the restoration of normoglycemia [40]–[42]. The significant upregulation of liver NUCB2 mRNA is possibly indicating a direct role for nesfatin-1 or other NUCB2 encoded peptides in liver physiology during food deprivation. One may speculate that this increase in liver NUCB2 suggests a potential tissue specific local role for NUCB2 peptides in the regulation of cellular gluconeogenic process in catabolic states. Further studies are warranted to determine the role of NUCB2 and nesfatin-1 on liver function, specifically in fed and fasted conditions.

The relative abundance of NUCB2 mRNA expression in goldfish adipose tissues may also be of particular importance. The role of adipose tissue in integrating metabolic activity and energy balance via adipokines has been thoroughly established [43]. Recent reports suggest that nesfatin-1 may play a role in adipocyte regulation in mice [44]. Circulating levels of nesfatin-1 and nesfatin-1 protein expression in adipose tissue is significantly elevated in high-fat-fed mice [44]. In addition, intracellular nesfatin-1 levels increase when adipose tissue is incubated in inflammatory cytokines [44]. Furthermore, the incubation of nesfatin-1 significantly increases the differentiation of preadipocytes into adipocytes [44]. It is possible that adipose tissue is a major source of circulating nesfatin-1 in goldfish and that nesfatin-1 has a role in adipose metabolism. However, the above observations from mammals cannot be applied to fish as they are poikilothermic organisms with different metabolic strategies. Whether, nesfatin-1 has similar conserved roles in teleost fishes is of great interest and should be investigated further.

Our *in vivo* studies in goldfish provide the first functional evidence for the appetite regulatory effects of nesfatin-1 in a non-mammalian vertebrate. Intraperitoneal injections of nesfatin-1 at a dose of 50 ng/g BW inhibited food intake by approximately 20% over the course of one hour. In mammals, a similar reduction of food intake has been observed with peripheral injections of nesfatin-1 over the first hour [1]. When nesfatin-1 was injected centrally at a dose as low as 0.5 ng/g BW we observed a striking 43% reduction in food intake over one hour. Although nesfatin-1 injected into the periphery has been shown to cross the blood brain barrier [45], [46] to cause the anorectic effect, Stengel et al., (2009) reported that peripheral injections of nesfatin-1 in mice reduced food intake, but the results were more readily observed through central injections. Peripherally administered nesfatin-1 might elicit its anorectic effects by acting on peripheral mechanisms such as gut motility or by acting at the appetite regulatory neurons after entering the brain. Although further studies are required to elucidate the mechanisms of actions and interactions of nesfatin-1 in the goldfish brain, our results clearly indicate an anorexigenic effect for the native form of nesfatin-1 in goldfish.

**Figure 9:**
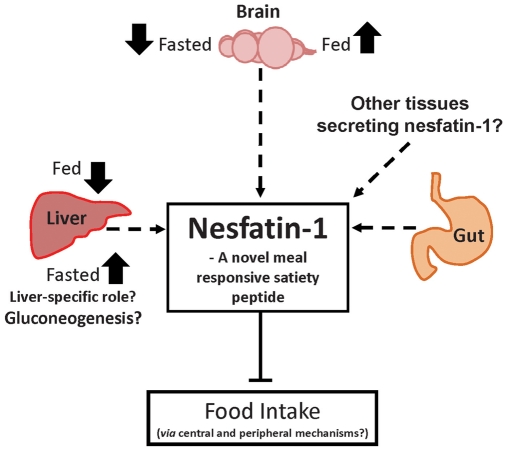
A summary on the distribution and functions of nesfatin-1 based on our findings in goldfish. NUCB2/nesfatin-1 is abundantly expressed in the brain, liver and gut. NUCB2 mRNA was upregulated in the brain of fed fish and in the liver of fasted fish, while NUCB2 mRNA was down-regulated in the brain of fasted fish and in the liver of fed fish (solid arrows). Although the major tissue source of circulating nesfatin-1 is currently unknown, we propose that nesfatin-1 is released in a meal responsive manner from several tissues, including the brain, liver and gut to inhibit feeding in fish via central and peripheral actions. Nesfatin-1 seems to have direct effects on the liver, possibly in regulating the metabolic processes, especially during food-deprivation (eg: gluconeogenesis?). Solid lines indicate direct known effects while dashed arrows indicate potential relationships.

Together, our results provide the first molecular and functional evidence for the wide presence and distribution of NUCB2 and anorectic effects of nesfatin-1 in non-mammalian vertebrates. This report furthers our current understanding of nesfatin-1 as a novel, meal responsive anorexigenic and metabolic peptide (**Figure 9**) with an evolutionarily conserved structure and function. Whether nesfatin-1 or other nesfatin peptides encoded in NUCB2 have biological actions other than the regulation of feeding has to be determined. The receptor(s) that mediates the actions of nesfatin-1 and the mechanisms of actions of nesfatin-1 remain unknown. The results of our studies set the stage for several lines of further investigations to better understand the functions and mechanisms of actions of NUCB2 and nesfatin-1, novel, endogenous biologically active peptides in vertebrates.

## Supporting Information

Table S1 Primers used for reverse transcription, 5′ and 3′ rapid amplification of cDNA ends, and quantitative real-time PCR of NUCB2 mRNA from the tissues of goldfish.
(TIF)

Figure S1 Nucleotide sequence of goldfish NUCB2 mRNA. Nesfatin-1 peptide region is underlined in black, nesfatin-2 is underlined in gray, and nesfatin-3 is underlined with dotted lines. The predicted cleavage sites required for processing are boxed.
(TIF)
