# Supplementary figures and images for: Molecular, Cellular and Physiological Evidences for the Anorexigenic Actions of Nesfatin-1 in Goldfish
Source: PLoS One. 2010 Dec 3;5(12):e15201. doi: 10.1371/journal.pone.0015201 (PMC2997068; doi:10.1371/journal.pone.0015201)

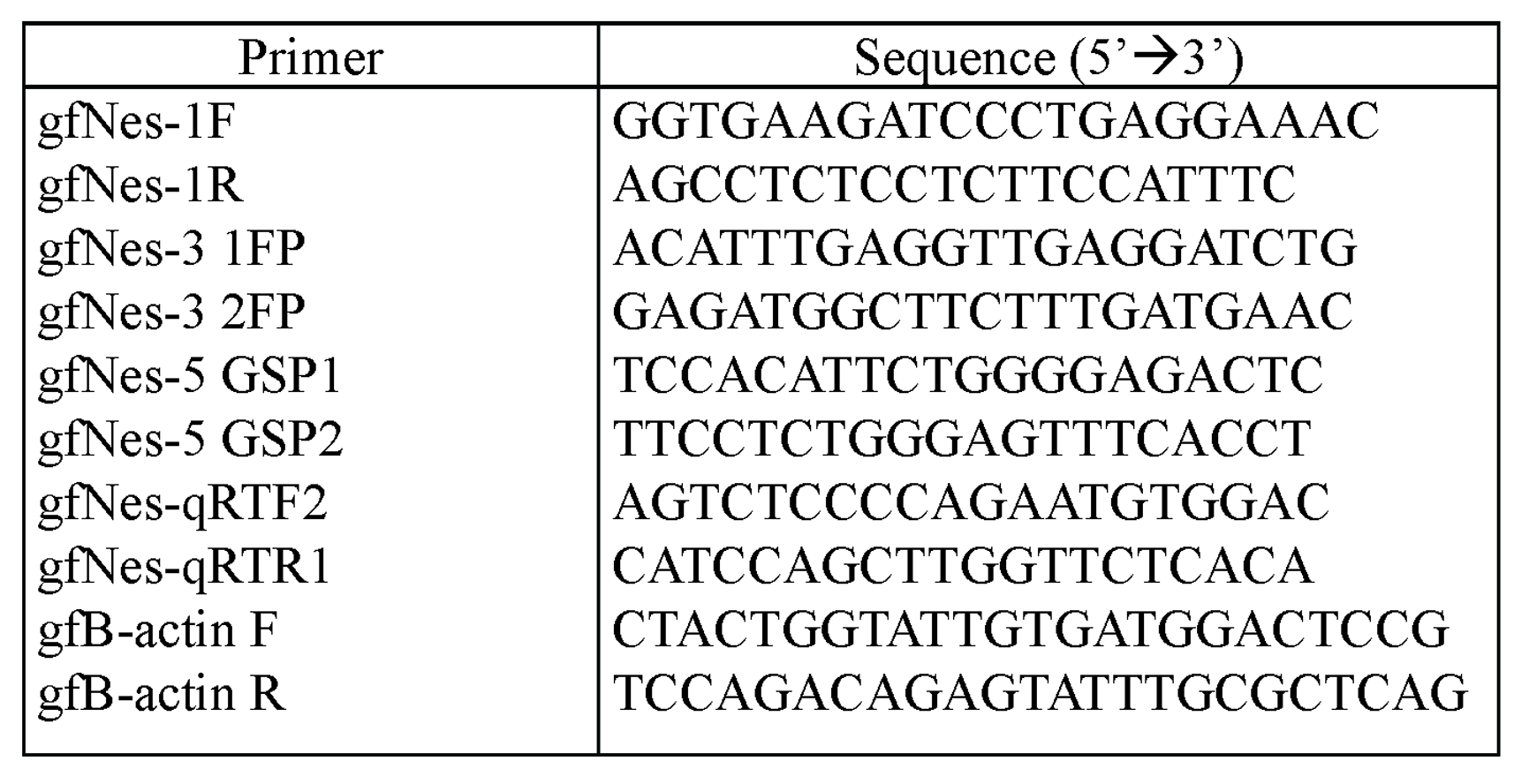

Supplement: Table S1 — Primers used for reverse transcription, 5′ and 3′ rapid amplification of cDNA ends, and quantitative real-time PCR of NUCB2 mRNA from the tissues of goldfish. (TIF) [file pone.0015201.s001.tif]

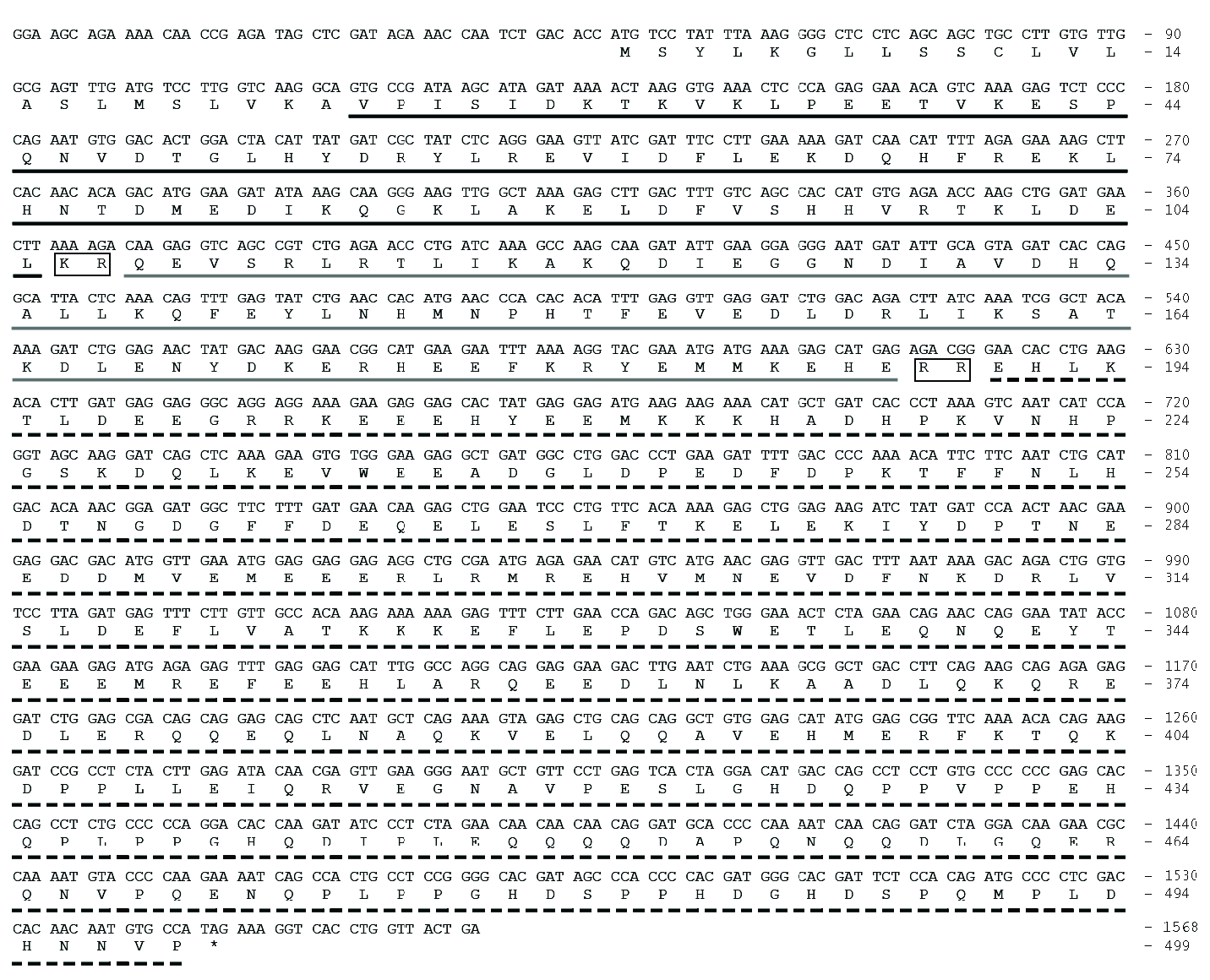

Supplement: Figure S1 — Nucleotide sequence of goldfish NUCB2 mRNA. Nesfatin-1 peptide region is underlined in black, nesfatin-2 is underlined in gray, and nesfatin-3 is underlined with dotted lines. The predicted cleavage sites required for processing are boxed. (TIF) [file pone.0015201.s002.tif]
